# It’s the Content That Counts: Longitudinal Associations between Social Media Use, Parental Monitoring, and Alcohol Use in an Australian Sample of Adolescents Aged 13 to 16 Years

**DOI:** 10.3390/ijerph18147599

**Published:** 2021-07-16

**Authors:** Anna Smout, Cath Chapman, Marius Mather, Tim Slade, Maree Teesson, Nicola Newton

**Affiliations:** 1 The Matilda Centre for Research in Mental Health and Substance Use, The University of Sydney, Sydney, NSW 2006, Australia; Cath.chapman@sydney.edu.au (C.C.); Tim.slade@sydney.edu.au (T.S.); maree.teesson@sydney.edu.au (M.T.); nicola.newton@sydney.edu.au (N.N.); 2 Turner Institute for Brain and Mental Health, Monash University, Melbourne, VIC 3800, Australia; 3 Sydney Informatics Hub, The University of Sydney, Sydney, NSW 2008, Australia; Marius.mather@sydney.edu.au

**Keywords:** social media, parental monitoring, alcohol, adolescent, longitudinal

## Abstract

(1) Background: More time spent on social media has been linked to increased alcohol use, with exposure to peer alcohol-related content on social media (content exposure) named as a critical factor in this relationship. Little is currently known about whether early content exposure may have lasting effects across adolescent development, or about the capacity of parental monitoring of social media use to interrupt these links. (2) Methods: These gaps were addressed in both cross-sectional and longitudinal contexts among a longitudinal sample of Australian secondary school students (*n* = 432) across the ages of 13–16. (3) Results: Evidence was found for links between social media use and alcohol use frequency in early development. Social media time at age 13 was significantly associated with concurrent alcohol use frequency. At age 13, alcohol use frequency was significantly higher among those who reported content exposure compared to those who reported no exposure. Longitudinally, the frequency of alcohol use over time increased at a faster rate among participants who reported content exposure at age 13. In terms of parental monitoring, no longitudinal effects were observed. However, parental monitoring at age 13 did significantly reduce the concurrent relationship between alcohol use frequency and content exposure. (4) Conclusion: The impact of social media content exposure on alcohol use in adolescence may be more important than the time spent on social media, and any protective effect of parental monitoring on content exposure may be limited to the time it is being concurrently enacted.

## 1. Introduction

In Australia and globally, alcohol use is common and makes a significant contribution to the burden of disease and injury, particularly amongst young people [1,2]. Alcohol misuse is associated with a range of deleterious lifelong sequelae, including substance dependence, mental disorders, suicidality, antisocial behavior, heart conditions, and cancer [3,4]. The legal age of purchase of alcohol in Australia is 18 years [5], and the average age of initiation of alcohol use is 16.2 years [6]. Early initiation of alcohol use has been associated with more frequent and risky patterns of substance use across development, an increased likelihood of developing substance dependence and the emergence of mental health disorders in adulthood [7,8,9]. The discernment of modifiable factors and phenomena that contribute to the engagement in and onset of alcohol use during adolescence is therefore critical.

The growth of social media use in the past decade has rapidly expanded the scope of social influence [10]. The influence of social media is said to surpass that of traditional media due to the fact that peers generate much of the content and are viewed as more relatable to users than celebrities [11]. Rates of social media use are highest among young people [10,12] with the vast majority (97%) of Australian teens aged 14–17 reportedly using social media [13]. On average, Australian teens use four different social media services [14] and spend more than three hours per day on their favored sites [12]. In response to such widespread use, the impact of social media use on adolescent well-being has become a topic of extensive scientific debate [15]. Though time spent on social media, referred to as social media time herein, has been linked with a range of adverse outcomes in adolescence (including depression and an increased frequency of alcohol use [16,17,18]), results from a series of recent reviews demonstrate that the picture is more complex [15,19,20,21]. To date, negative associations between social media use and well-being have been small in size, and largely based on correlational analysis [19,20,21]. In response, the literature calls for longitudinal examination of, and consideration of mechanisms underlying, such associations in the pursuit of delineating iatrogenic outcomes of social media use [15,19,20,21].

Exposure to peer-generated content depicting risky substance use, referred to as content exposure herein, is one such mechanism posited to underly the relationship between social media time and an increased frequency of alcohol use [10,22,23]. Substance-related content most commonly comprises pictures or text depicting alcohol consumption, being drunk, or hungover, usually portrayed in a positive light [24,25]. Exposure to content on social media depicting substance use is common [26], and is said to bias and inflate normative perceptions of observed behaviors [27,28]. Moreover, qualitative studies have found that adolescents interpret online displays of alcohol use as reliable depictions of actual use, and that younger adolescents (age 12–14) as compared to older adolescents (age 15–18) are particularly susceptible to these beliefs [29]. As such, the influence of content exposure on an earlier engagement in alcohol use warrants further exploration. To our knowledge, only one study to date has delineated associations between social media time, content exposure, and alcohol use among adolescents. This study found that while exposure to peer-generated content displaying risky substance use at age 15 predicted tobacco and alcohol use outcomes at age 16, the frequency of social media use alone did not [22]. It is currently unknown whether such associations may take hold even earlier in development, and whether early exposure may have lasting effects. 

Given the range of adverse short- and long-term health outcomes linked to the early initiation of alcohol use, identifying early influences, as well as the means through which alcohol uptake may be delayed, remain public health priorities. The parental monitoring of *general* child and adolescent media use has been associated with a reduction in associated adverse outcomes such as aggression, online harassment, poorer sleep, and academic performance [30,31,32]. Importantly, however, the efficacy of parental monitoring in ameliorating negative effects of general media use has been found to vary according to monitoring style [33,34]. In this context, distinctions are made between active (critically discussing media content with children) and restrictive (placing limits around the amount of media time or type children are permitted to engage with) monitoring styles [33,35]. Whether any parental monitoring of *social* media use may serve to mediate relationships between social media use and related adverse outcomes (such as alcohol use) remains currently untested and an important line of enquiry given (a) the modifiable nature of parenting practices and (b) the low reported rates of parental monitoring of social media use. Indeed, survey data collected in 2017 found that according to both parents and adolescents (aged 14–17), 60% of Australian parents reportedly never monitored their child’s use of social media within the previous 12 months [11].

### Aims of the Present Study

Among a longitudinal Australian sample of middle school students (age 13–16), the current study aimed to investigate early relationships between social media use and alcohol use, distinguishing between time spent on social media (social media time) and exposure to peer-generated content on social media depicting risky substance use (content exposure). We also aimed to examine the impact of the parental monitoring of social media use on these relationships. Specifically, we aimed to examine:

(1) Concurrent associations between social media time, content exposure, and frequency of alcohol use at age 13.
(2) Whether content exposure moderated the association between social media time and frequency of alcohol use at age 13.
(3) Longitudinal associations between social media use (time and content exposure) at age 13, and frequency of alcohol use over time (up to age 16).
(4) Whether adolescent perception of parental monitoring of social media use at age 13 served to moderate any of the aforementioned relationships.

## 2. Methods

### 2.1. Participants and Procedure

The present sample comprised participants allocated to the control group of a cluster-randomized controlled trial designed to assess the effectiveness of a combined universal and selective school-based prevention program targeting substance misuse and related harms, a detailed description of which has been published elsewhere [36]. The 26 participating Australian secondary schools were a mixture of public and independent, single-sex and co-educational. Seven schools were randomly allocated to the control group where participants received no intervention, and 527 students with parental and self-consent completed baseline surveys (69.8% female; mean age: 13.4 years, *SD* = 0.44). One control school did not provide social media data due to ethical restrictions, resulting in a final sample for the present study of 441 students from six schools (five independent and one public). This study conducted secondary analyses on data collected at baseline, and at 6, 12, 24, and 36 months post-intervention, when students were aged 13, 13.5, 14, 15, and 16 years. Starting in 2012, self-report questionnaire data were collected at each time point in a classroom setting, supervised by a teacher or research team member. 

### 2.2. Measures

Demographics: Participants provided their age and gender at each wave of data collection.

Alcohol use frequency: Alcohol use frequency was assessed using the item ‘how often did you have a standard alcoholic drink of any kind in the past 6 months?’. A standard alcoholic drink is defined in Australia as a drink that contains 10 g of alcohol. Responses to these items fell into six categories (*Never/Less than monthly/Monthly/2–3 times a month/Weekly/Daily or almost daily*). This variable was treated as a continuous variable, recoded to number of days drinking per month (*never* = 0, *less than monthly* = 0.5, *monthly* = 1, *2–3 times a month* = 2.5, *weekly* = 4, *daily* = 30). 

Social media use and parental monitoring: Three items were adapted from the 17th Annual ‘back-to-school survey’ conducted by The National Centre on Addiction and Substance Abuse at Columbia University [37] to assess social media use and parental monitoring. To measure social media time, participants provided a free entry numeric response to the item ‘how many minutes do you spend on Facebook, Myspace and other social networking sites in a typical day?’. Responses greater than 12 h per day were truncated to 12 h. The item ‘Do you see pictures of kids drunk, passed out or using drugs on these sites?’ was used to measure content exposure, and the item ‘Does your parent monitor your use of social networking sites?’ was used to measure perceived parental monitoring, both of which had dichotomous (*Yes/No*) response options.

### 2.3. Covariates

Gender: Differential effects of gender have been observed in the frequency of both social media and substance use. Namely, male adolescents are more likely to drink than their female counterparts [38,39], and females report more frequent engagement with social media sites than males [32,40,41]. As such, gender was entered as a covariate in the models.

Personality: Research targeting the prevention of substance misuse has proposed four personality-related pathways that are linked with a heightened vulnerability to experiencing substance use problems (impulsivity, sensation seeking, hopelessness, and anxiety sensitivity [42,43]). Significant, positive correlations have also been found between increased social media use, impulsivity, and sensation seeking, as well as between social media use and symptoms of depression and anxiety [16,44,45]. Therefore, scores on each of the four personality traits, as measured by the Substance Use Risk Profile Scale (SURPS) [46], were entered as covariates in the models. The reliability and validity of the SURPS has been established among youth populations in the Netherlands, the United States, Canada, and Australia [43,46,47,48,49,50].

### 2.4. Missing Data

Missing data occurred when a participant was absent for a survey, failed to answer a question, or provided invalid responses. In the present sample, 94% of participants completed baseline and at least two follow-up surveys. In cross-sectional analyses, missing data were deleted listwise from the model. For the longitudinal analyses, we retained all available observations of each participant in the model.

### 2.5. Statistical Analysis

Analyses were conducted using R version 3.6 [51]. Cross-sectional relationships were examined using multiple linear regression analyses controlling for gender and baseline SURPS subscale scores as covariates. SURPS scores were standardized before being included in the regression models. Categorical predictors including gender, content exposure, and parental monitoring were dummy coded (0: male/no/absent, 1: female/yes/present). Cross-sectional analyses examined the relationship between predictors of interest and alcohol use frequency at age 13 using models that contained social media time and content exposure, with and without parental monitoring and interaction terms.

Longitudinal relationships were analyzed using mixed linear regression models. Time was modeled as a linear term, coded as the number of years after baseline. Random intercepts were estimated for each participant to account for the correlation between their measurements. Random school effects were not modeled because initial analyses showed low intracluster correlations (<10%) at the school level [52]. As with the cross-sectional analyses, these analyses were run controlling for gender and baseline SURPS subscale scores. Separate models were run to address each research aim, adding parental monitoring predictors along with interactions that related to the research aims.

## 3. Results

The sample consisted of 441 participants from six schools. Characteristics of the sample at baseline are shown in Table 1.

### 3.1. Descriptive Statistics

Means, standard deviations, and percentages corresponding to alcohol use frequency and variables relating to social media use are shown in Table 2. On average, days drinking per month was low in this sample—less than one day per month, with the exception of time 5 (16 years of age). Hours spent per day on social media and the occurrence of content exposure increased across each time point, while parental monitoring decreased.

### 3.2. Cross-Sectional Results

Cross-sectional relationships between social media variables are shown in Table 3.

Model 1: Social media time and content exposure. Controlling for gender and SURPS, when alcohol use frequency was regressed on social media time and content exposure, increased social media time (and not content exposure) was associated with an increased frequency of drinking at age 13 (*b* = 0.03, 95% CI [0.01, 0.06], *p =* 0.021).

Model 2: Moderation by content exposure. The content exposure × social media time interaction term was added to model 1 and was statistically significant at age 13 (*b* = 0.08, 95% CI [0.03, 0.14], *p* = 0.003). 

Model 3: Moderation by parental monitoring (social media time). There was no evidence of an interaction between social media time and parental monitoring on the frequency of alcohol use at age 13.

Model 4: Moderation by parental monitoring (content exposure). Controlling for social media time, the interaction between content exposure and parental monitoring was significant at age 13 (*b =* −0.15, 95% CI [−0.28, −0.02], *p* = 0.308).

### 3.3. Longitudinal Results

Longitudinal relationships between social media use and frequency of drinking are shown in Table 4. Frequency of alcohol use significantly increased over the five time points (Model 5: *b* = 0.30, 95% CI [0.22, 0.39], *p* < 0.001). 

Model 6: Social media time and drinking. There was no significant association between social media time at baseline and alcohol use frequency over time (*b* = 0.10, 95% CI [0.01, 0.21], *p* = 0.075).

Model 7: Content exposure. There was evidence of an interaction between content exposure at baseline and frequency of alcohol use over time, such that the frequency of alcohol use increased at a faster rate among participants exposed to images of alcohol use at baseline (*b* = 0.29, 95% CI [0.04, 0.53], *p* = 0.024), after controlling for social media time.

Model 8: Parental monitoring and social media time. Controlling for social media time, there was no evidence of an interaction between parental monitoring and time (years) on frequency of drinking over the course of the study (*b =* 0.07, 95% CI [−0.15, 0.28], *p* = 0.542).

Model 9: Parental monitoring and content exposure. Controlling for content exposure, there was no evidence of an interaction between parental monitoring and time (years) on frequency of drinking over the course of the study (*b =* 0.07, 95% CI [−0.13, 0.27], *p =* 0.504).

## 4. Discussion

The present study examined the cross-sectional and longitudinal relationships between social media use (distinguishing between time spent on social media and exposure to peer-generated content depicting risky substance use), alcohol use, and parental monitoring among a sample of Australian adolescents aged 13–16 years. Results are discussed below within the context of developmental risk/exposure and implications for parenting practices.

First, with regard to objective 1, time spent on social media at age 13 was significantly associated with a concurrent increased frequency of alcohol use, over and above covariates (gender and four personality vulnerability factors associated with substance misuse). However, seeing images of others drunk, passed out, or using drugs (content exposure) was not significantly associated with alcohol use frequency at this age. Nonetheless, content exposure did change the way social media time associated with alcohol use frequency (objective 2). Although the effect size is small, this significant interaction indicates that where more social media hours per day is related to an increased frequency of alcohol use, this relationship occurs *only* when adolescents are exposed to images of peers engaging in substance-related risk behaviors. When adolescents reported no exposure to such content, the relationship between social media time and alcohol use frequency was essentially absent. These outcomes support the idea that content exposure may warrant more attention than social media time when it comes to prevention of alcohol use in early adolescence. 

Furthermore, with regard to objective 3, content exposure at age 13 emerged as the only variable in this study to have longitudinal associations with alcohol use frequency. Content exposure at age 13 was significantly associated with a faster rate of increase in alcohol use frequency up to age 16, after controlling for social media time and related covariates (model 7). This pattern of outcomes aligns with those of Huang et al. (2014), who employed social network analyses to examine the influence of 15-year old adolescent Facebook and MySpace behaviors on drinking and smoking outcomes six months later. While neither the frequency of social media use nor the number of reported friends on social media predicted risk behaviors six months later, those with more friends who posted content depicting ‘partying’ or alcohol use online at age 13 were significantly more likely to have smoked tobacco or consumed alcohol six months later. Our results extend this finding, suggesting that any effects of early content exposure may persist longer than six months and throughout formative adolescent years. 

Finally, in terms of parental monitoring (objective 4), the significant interaction between content exposure and parental monitoring at age 13 provides some support for the role of concurrent parental monitoring in moderating positive relationships between content exposure and alcohol use frequency (model 4). Notably, however, this effect size was small, and parental monitoring appeared to have no significant impact on associations between social media variables and alcohol use frequency over time (models 8 and 9). This absence of a longitudinal effect is consistent with the literature, e.g., [39,40], and suggests that any protective effect of parental monitoring on content exposure may be limited to the time it is being concurrently enacted. 

There are limitations to this study that warrant mention. First, the present sample was drawn primarily from independent schools with a higher proportion of females, which may limit some variability in the sample. This is particularly relevant given rates of alcohol use and related harms are traditionally lower in females compared to males [5,39]. Future studies may wish to ensure they recruit schools of diverse socioeconomic positions in order to increase generalizability. Small effect sizes and nonsignificant findings may reflect the lack of variability (or power) to detect effects. 

Second, the presence of some missing data due to participant attrition throughout follow-up surveys may limit the reliability of the longitudinal findings. With fewer observations, there may have been a lower power to detect associations at later time points. That said, 94% of this sample completed baseline and at least two follow-up surveys, and the longitudinal models used included all available observations for each participant. Thirdly, limitations exist around the specificity of some questionnaire items. Specifically, the dichotomous nature of the content exposure and parent monitoring items, and the lack of items assessing amount or style of parental monitoring. Additionally, the wording of the content exposure item implies a high level of alcohol use, while any level of use (i.e., using alcohol without being drunk or passed out) could be considered risky among adolescents aged 13–16. The reliance on child self-report of parental monitoring of social media use is also notable because it is possible that this may not be a true depiction of parental monitoring occurrence. For instance, some parents may employ covert monitoring techniques of which children are unaware. Assessing parental monitoring using subjective, objective, qualitative, and quantitative measures from the perspective of both the adolescent and parent in future research would be beneficial to improve the specificity of recommendations for parents around adolescent social media monitoring. 

Despite these limitations, the findings from this study have important implications for future research. First, however, in light of the emphasis placed on the impact of social media use during adolescence, it is appropriate at this juncture to emphasize that the predictors of focus in the present paper were prioritized because they are modifiable and occur early in adolescent development. They were not selected because they are considered a silver bullet in preventing substance use across development. The small-to-moderate effect sizes observed likely reflect this notion, mirroring arguments put forward by Orben and Przybylski (2019) that conclusions about the potential impact of modifying any risk factor should be put in the context of the size of the observed effect and the full array of risks for harmful outcomes.

## 5. Conclusions

Nonetheless, the outcomes of the present study contribute longitudinal evidence to a growing research base that calls for a more nuanced conceptualization of social media use when examining its impact on adolescent well-being and development. Our findings support the view that social media time (alone) may not be so pertinent to consider in affecting the frequency of alcohol use in adolescence. Rather, it is exposure to peer-generated content depicting risky substance use on social media that may have lasting effects, and parental monitoring of social media use may have some efficacy in mitigating these links in early adolescence. It follows that active parental monitoring (targeting the type of content consumed) may be superior to restrictive monitoring (limiting time spent) in this pursuit—a hypothesis that warrants further exploration. Keeping in mind that for each year the uptake of alcohol use is delayed in adolescence, the chance of developing an alcohol use disorder in adulthood is reduced by almost 10% [53], identifying and developing our opportunities for early prevention remains critical.

## Figures and Tables

**Table 1:** Characteristics of the study sample at baseline.

Overall	
*N* schools	6
–Independent schools	5
*N* participants	441
–In independent school	432/441 (98.0%)
Age [years, *M* (SD)]	13.4 (0.439)
Female	308/441 (69.8%)
Born in Australia	393/441 (89.1%)
Ever had a full drink of alcohol	63/440 (14.3%)

**Table 2:** Descriptive statistics for alcohol use frequency and social media use variables.

Time	Days Drinking per Month [*M* (SD)]	Social Media Time (Hours per Day) [*M* (SD)]	Content Exposure [*n* (%)]	Parental Monitoring [*n* (%)]
Baseline (age 13)	0.156 (1.46)	0.765 (1.02)	104/414 (25.1%)	198/414 (47.8%)
Time 2 (age 13.5)	0.420 (3.09)	0.932 (1.24)	100/338 (29.6%)	145/338 (42.9%)
Time 3 (age 14)	0.210 (1.56)	0.956 (1.26)	149/374 (39.8%)	143/374 (38.2%)
Time 4 (age 15)	0.610 (2.74)	1.17 (1.47)	209/355 (58.9%)	120/354 (33.9%)
Time 5 (age 16)	1.22 (3.74)	1.35 (1.54)	182/311 (58.5%)	77/311 (24.8%)

**Table 3:** Cross-sectional relationships between social media use variables and alcohol use frequency (typical days drinking per month).

Predictors (*n* = 1727)	*b*	95% CI	*p*
Model 1: Social media time and content exposure			
Social media time	0.03	0.01 to 0.06	0.021 *
Content exposure	0.04	−0.02 to 0.10	0.224
Model 2: Social media time × content exposure			
Social media time	0.01	−0.03 to 0.04	0.763
Content exposure	−0.05	−0.14 to 0.04	0.268
Social media time × Content exposure	0.08	0.03 to 0.14	0.003 *
Model 3: Social media time × Parental monitoring			
Social media time	0.06	0.02 to 0.09	0.001
Parental monitoring	0.00	−0.06 to 0.07	0.898
Social media time × Parental monitoring	−0.05	−0.10 to 0.00	0.065
Model 4: Content exposure × Parental monitoring			
Social media time	0.03	0.00 to 0.06	0.043 *
Content exposure	0.10	0.02 to 0.18	0.020 *
Parental monitoring	0.00	−0.06 to 0.06	0.890
Content exposure × Parental monitoring	−0.15	−0.28 to −0.02	0.020 *

Note: All models controlled for gender and SURPS subscale scores. *b*: unstandardized regression coefficient. 95% CI: 95% confidence interval for the regression coefficient. * indicates significant at the *p* < 0.05 level.

**Table 4:** Longitudinal relationships between social media use and alcohol use frequency (typical days drinking per month).

Predictors (*n* = 1727)	*b*	95% CI	*p*
Model 5: Time since baseline			
Time since baseline	0.30	0.22 to 0.39	<0.001 *
Model 6: Social media time			
Time since baseline	0.29	0.16 to 0.42	<0.001 *
Social media time	−0.09	−0.29 to 0.10	0.355
Time × Social media time	0.10	−0.01 to 0.21	0.075
Model 7: Content exposure			
Time since baseline	0.30	0.18 to 0.42	<0.001 *
Content exposure	−0.04	−0.50 to 0.41	0.854
Social media time	0.05	−0.10 to 0.20	0.545
Time × Content exposure	0.29	0.04 to 0.53	0.024 *
Model 8: Parental monitoring (controlling for social media time)			
Time since baseline	0.33	0.18 to 0.48	<0.001 *
Parental monitoring	−0.02	−0.41 to 0.36	0.902
Social media time	0.02	−0.13 to 0.17	0.769
Time × Parental monitoring	0.07	−0.15 to 0.28	0.542
Model 9: Parental monitoring (controlling for content exposure)			
Time since baseline	0.32	0.18 to 0.46	<0.001 *
Parental monitoring	−0.01	−0.37 to 0.36	0.970
Content exposure	0.22	−0.10 to 0.54	0.178
Time × Parental monitoring	0.07	−0.13 to 0.27	0.504

Note: All models controlled for gender and SURPS subscale scores. *b*: Unstandardized regression coefficient. 95% CI: 95% confidence interval for the regression coefficient. * indicates significant at the *p* < 0.05 level.

## Data Availability

Data are not available, due to ethical restrictions.
